# Identification of the *FLA* Gene Family in Soybean and Preliminary Functional Analysis of Its Drought-Responsive Candidate Genes

**DOI:** 10.3390/genes16121425

**Published:** 2025-11-29

**Authors:** Jiyue Zhang, Lina Yang, Jingxuan Dou, Cong Wang, Zhengpei Yao

**Affiliations:** 1 Xinjiang Key Laboratory for Ecological Adaptation and Evolution of Extreme Environment Organisms, College of Life Sciences, Xinjiang Agricultural University, Urumqi 830052, China; zjyzhang995@163.com; 2 College of Life Sciences, Xinjiang Agricultural University, Urumqi 830052, China; yanglina_2025@163.com (L.Y.); doujingxuan22@163.com (J.D.); 3 College of Agriculture, Xinjiang Agricultural University, Urumqi 830052, China; soybean2020@126.com

**Keywords:** soybean, gene family, FlAs, drought

## Abstract

**Background/Objectives:** Fasciclin-like arabinogalactan proteins (FLAs) are critical components of the plant cell wall, playing vital roles in development and abiotic stress responses. However, a systematic genome-wide analysis of the FLA family in soybean (*Glycine max* L.), a major legume crop susceptible to drought, is lacking. This study aimed to comprehensively identify *GmFLA* members and investigate their potential functions in drought tolerance. **Methods:** We identified *GmFLA* genes via BLASTP (v2.16.0) and Hidden Markov Model (HMM) searches against the soybean genome. Subsequent analyses encompassed their physicochemical properties, chromosomal distribution, gene structure, phylogenetic relationships, conserved domains, cis-acting elements, and subcellular localization. Drought-responsive candidates were screened using a Gene Expression Omnibus (GEO) dataset, and their expression profiles were validated under drought stress using quantitative real-time PCR (qRT-PCR). **Results:** A total of 55 *GmFLA* genes were identified and unevenly distributed across 14 chromosomes. Most genes featured a single-exon structure and contained a conserved Fasciclin domain, with predicted localization primarily to the chloroplast. Phylogenetic analysis grouped them into three distinct subclasses with Arabidopsis homologs, suggesting lineage-specific expansion. Promoter analysis revealed an abundance of stress- and hormone-related cis-elements. Expression analysis identified five candidate genes (*GmFLA*5, *GmFLA*15, *GmFLA*40, *GmFLA*47, and *GmFLA*54) that showed tissue-specific expression changes under drought treatment. **Conclusions:** This study provides the first comprehensive genomic characterization of the *GmFLA* gene family and identifies candidate *GmFLA*s with drought-responsive expression patterns. Our findings establish a foundation for future functional research to investigate their potential roles in soybean drought response. Furthermore, these candidates serve as potential targets for further investigation in strategies aimed at improving soybean drought tolerance.

## 1. Introduction

Plant cells adapt to environmental changes, respond to external stimuli, and maintain homeostasis through intricate intercellular communication and interactions [1]. This process relies on signal transmission across the cell wall. As a dynamic and complex structure, the cell wall not only provides mechanical support and protection but also directly participates in signal transduction, cell communication, and immune defense [2]. Critically, the cell wall is not a static barrier but is actively remodeled under drought stress. This remodeling manifests in various ways: for instance, drought inhibits the biosynthesis of primary cell wall components like pectin and hemicellulose in cotton fibers, suppressing the expression of key biosynthetic genes such as galacturonosyltransferase (*GhGAUT*) and xylosyltransferase (*GhXXT*) [3]. Concurrently, studies in Arabidopsis have shown that drought stress alters the expression and activity of xyloglucan-modifying enzymes such as xyloglucan endotransglucosylase/hydrolases (*XTHs*), which remodel the cellulose–xyloglucan network and influence cell wall mechanical properties and water retention capacity [4]. These changes collectively influence cell wall mechanical properties and water retention capacity. Hydroxyproline-rich glycoproteins (HRGPs) are a widely distributed class of proteins within the cell wall, playing crucial roles in plant growth and development [5] as well as disease resistance [6]. Based on differences in glycosylation levels, the HRGP superfamily can be classified into three categories: arabinogalactan proteins (AGPs), proline-rich proteins (PRPs), and extended proteins (EXTs) [7].

Arabinogalactan proteins (AGPs) are a class of highly glycosylated glycoproteins, with their glycosyl side chains accounting for over 90% of their molecular weight. Most are GPI-anchored to the plasma membrane [8]. AGPs are widely distributed in the cell walls, plasma membranes, and extracellular matrix of higher plants [9], participating in regulating various physiological processes such as somatic embryogenesis, cell proliferation, pollen tube growth, and hormone signaling [10]. Based on protein structural features, AGPs can be further classified into classical AGPs, AG peptides, chimeric AGPs (CAGPs), and non-classical AGPs [11,12]. Among these, fasciclin-like AGPs (FLAs) within chimeric AGPs have garnered significant attention due to their incorporation of 1–2 fasciclin domains with adhesive functions [13]. Notably, FLAs are implicated in cell adhesion, intercellular communication, and abiotic stress responses [4]. Current understanding of *FLA* genes in drought response among legumes is still developing. While transcriptomic studies in model legumes like Medicago truncatula have identified various drought-responsive genes, the specific mechanisms and functional roles of FLAs in this process require further elucidation.

Fasciclin-like arabinogalactan proteins (FLAs) constitute a well-defined AGP subfamily. Beyond the typical AGP glycosylation region, they contain 1–2 FAS domains [14] and predominantly possess an N-terminal signal peptide and a C-terminal GPI anchor signal. The *FLA* gene family has been identified in various plant species, with species-specific variations in the number of members. Examples include *Arabidopsis thaliana* (L.), *Oryza sativa* L., *Triticum aestivum* L., *Populus trichocarpa* Torr. & Gray, American black poplar (*Populus deltoides* Marshall), *Salix suchowensis* W.C. Cheng in S.Y. Jin, cotton (*Gossypium hirsutum* Linn), Chinese cabbage (*Brassica rapa* ssp. chinensis), and tomato (*Solanum lycopersicum* L.), with 21, 27, 34, 35, 40, 46, 19, 36, and 24 *FLA* members, respectively [15,16,17,18,19,20,21,22]. FLAs are increasingly recognized not only as structural components but also as potential contributors to cell wall integrity sensing and stress signaling, suggesting their possible role in connecting cell wall biology with stress physiology. Fasciclin-like arabinogalactan proteins (FLAs) are key glycoproteins regulating cell wall development and cellulose deposition, broadly influencing crop fiber quality and stem mechanical properties [23,24]. Research indicates that cotton *GhFLA*1 regulates fiber initiation and elongation [25]; Arabidopsis *AtFLA*11/12 participates in stem biomechanical regulation [26], *AtFLA*3 influences pollen development [27], while *AtFLA*1/4/18 mediate root development and ABA signal transduction in stress responses [15,28,29]. Furthermore, this family’s involvement in abiotic stress response is evidenced in other species: in tomato, *SlFLA*1 and *SlFLA*3 were significantly induced by drought stress [22], while conversely, in potato, *StFLA*4 acted as a negative regulator of drought tolerance [30]. Separately, the banana *MaFLA*27 gene was shown to confer cold tolerance by modulating cell wall remodeling [4].

As a major food and oil crop and model legume, soybean production is frequently constrained by drought stress [31,32]. Although *FLA* genes have been extensively studied across multiple species, their systematic identification in soybean and functional roles in drought response remain unclear. This study aims to deepen understanding of this gene family in soybean and lay the foundation for future functional studies of soybean *FLA* genes in drought response. To this end, we conducted a genome-wide identification of the *FLA* gene family in soybean, analyzing its phylogenetic relationships, gene structures, conserved motifs, and promoter cis-acting elements. By integrating public transcriptomic data with qRT-PCR experiments, we preliminarily screened and validated several drought-response candidate genes. This establishes a theoretical foundation for subsequent investigations into the functional mechanisms of soybean *FLA* genes in drought tolerance and growth development.

## 2. Results

### 2.1. Identification and Nomenclature of the FLA Gene Family

This study employed an integrated strategy to systematically identify members of the FLA gene family in the soybean (*G. max*) genome. First, homology-based BLASTP alignment was performed using Arabidopsis FLA protein sequences as queries (E-value < 1 × 10^−5^), combined with a Hidden Markov Model (HMM) search for the conserved Fasciclin domain (PF02469) from the Pfam database (E-value < 1 × 10^−5^). The candidate sequences from both approaches were merged and subjected to rigorous online validation using the NCBI Conserved Domain Database. This validation confirmed that all 55 non-redundant genes contained a complete, typical Fasciclin domain (domain E-value < 0.01 and coverage > 60%). Based on their physical locations across soybean’s 20 chromosomes, these genes were sequentially named GmFLA1 through GmFLA55.

### 2.2. Chromosomal Distribution and Gene Localization

**Figure 1:**
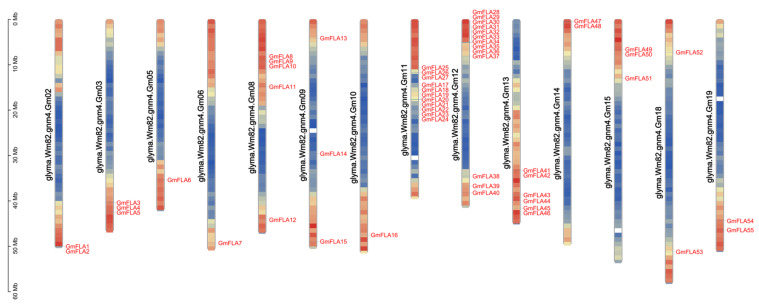
Chromosomal distribution of the soybean *FLA* gene family. Chromosome labels are positioned on the left side of the bar chart, gene names are highlighted in red, and the scale bar is located on the left.

Chromosomal distribution analysis revealed (Figure 1) that the 55 *GmFLA* genes are not evenly distributed across all chromosomes but are concentrated on 14 chromosomes. Among these, distribution on chromosomes 11 and 12 is particularly concentrated. Analysis indicates no significant correlation between the number of genes on each chromosome and its physical size.

### 2.3. Analysis of Physicochemical Properties of Encoded Proteins

A systematic analysis of the physicochemical properties of *GmFLA proteins* was conducted (Table 1). The amino acid lengths of family members ranged from 206 (*GmFLA*13) to 454 (*GmFLA*39), with most concentrated between 250 and 450 amino acids; their predicted molecular weights ranged from approximately 24 kDa (*GmFLA*13) to 50 kDa (*GmFLA*39), with most falling between 25 and 50 kDa. The isoelectric point (pI) distribution was broad, spanning from 4.53 (*GmFLA*13) to 9.48 (*GmFLA*42), with most members exhibiting pI values between 5.0 and 9.5. The average instability index is 43.5, though most members have values below 40, suggesting these proteins may exhibit high stability. Lipid indices are generally high (average 94.3, with most distributed between 80 and 105), reflecting a strong hydrophobic tendency across the family. Further hydrophilicity/hydrophobicity analysis revealed that 40% of members exhibited hydrophobic properties, notably *GmFLA*19 (0.263), *GmFLA*14 (0.189), and *GmFLA*13 (0.178); while the remaining 60% exhibited hydrophilicity, such as *GmFLA*32 (−0.331), *GmFLA*27 (−0.308), and *GmFLA*5 (−0.235).

Additionally, subcellular localization predictions indicate that members of this family exhibit diverse potential localizations: 26 members are predicted to localize to chloroplasts, while the remaining members localize to the cell wall, vacuole, and plasma membrane. It should be noted that these predictions are based solely on amino acid sequences and may not fully reflect the complex post-translational modification processes that ultimately determine FLA protein localization. As recently revealed by studies on Arabidopsis FLA proteins, the final localization of FLA proteins may be jointly regulated by GPI anchoring and AG glycosylation. These two mechanisms collectively determine their cell surface localization and subsequent release to the cell wall [33]. Signal peptide prediction results indicate that, except for *GmFLA*11, *GmFLA*13, *GmFLA*20, *GmFLA*25, and *GmFLA*42, all other members contain signal peptides. This strongly supports that the vast majority of *GmFLA*s enter the secretory pathway and function at the cell surface or within the cell wall matrix.

### 2.4. Structural and Phylogenetic Analysis of the FLA Gene Family in Soybean

**Figure 2:**
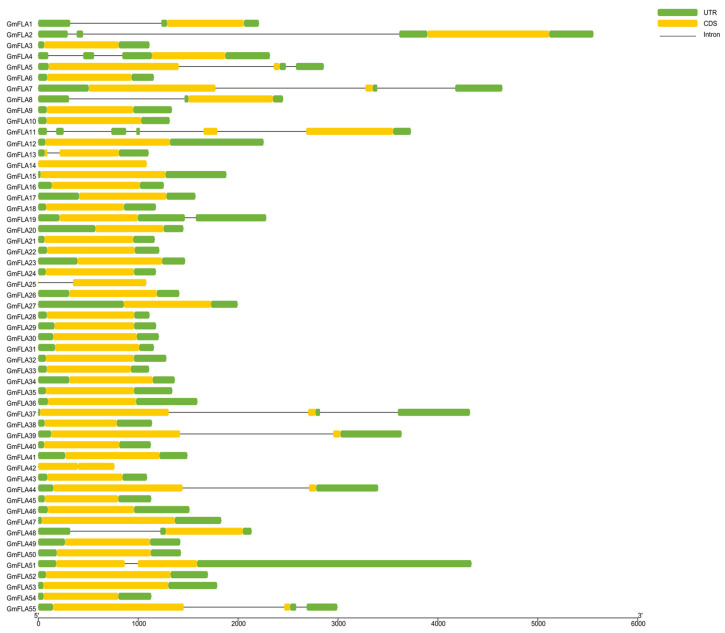
Exon–intron structure of the soybean *FLA* gene family: Untranslated regions (UTRs) are marked with green bold boxes, exons are indicated by yellow bold boxes, and introns are represented by black lines.

Our analysis indicates that the number of exons varies among members of this family, ranging from 1 to 6 (Figure 2). Notably, the majority of members (73%) contain a single exon, suggesting this is a characteristic feature of the family. Most *FLA* family members lack introns; only 16 members possess introns, and 10 of these have two or more introns. The lengths of these introns vary significantly across members and positions, ranging from tens of base pairs to several kilobases. This suggests that multi-intron structures may be retained by functional selection and could be associated with more complex alternative splicing regulation.

**Figure 3:**
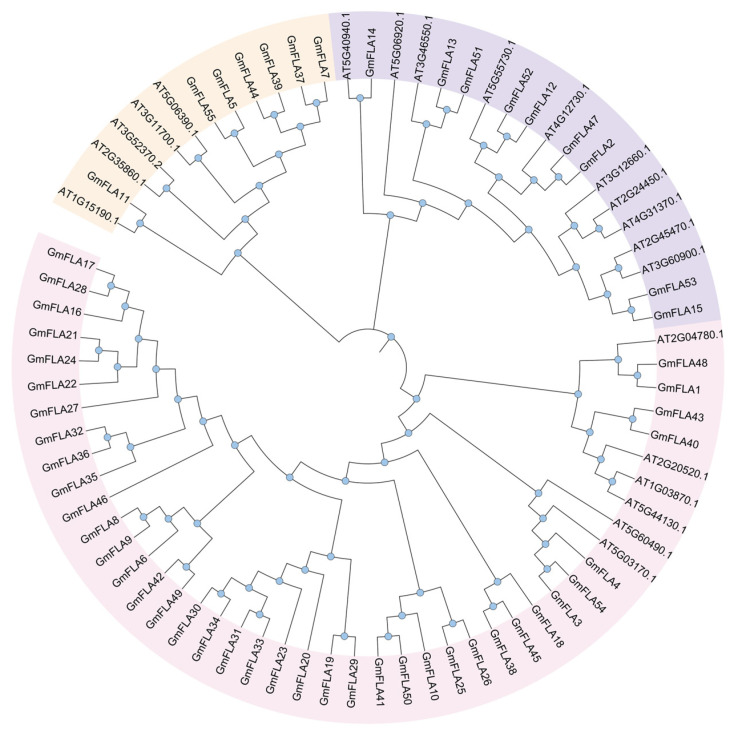
Phylogenetic Tree of the *FLA* Gene Family in Soybean and Arabidopsis.

To investigate the evolutionary relationships among the *FLA* gene families in soybean and Arabidopsis, we constructed a phylogenetic tree using MEGA 11 software (Figure 3). As shown, FLA proteins from these plants were grouped into three subclades based on homology. The smallest subclade contained 12 members (7 from the soybean *FLA* family), while the largest subclade comprised 45 members (39 from the soybean *FLA* family, accounting for 87% of the largest subclade). This subclade exhibited numerous soybean-specific branches independent of Arabidopsis. Soybean underwent extensive lineage-specific gene duplication events during evolution, exhibiting significantly higher expansion intensity than Arabidopsis. This provides a molecular basis for the adaptive evolution of legumes. Future research should focus on elucidating the specific regulatory mechanisms of these branched genes in stress responses and other potential biological processes.

### 2.5. Analysis of the Conserved Domains and Motifs in the Soybean FLA Gene Family

**Figure 4:**
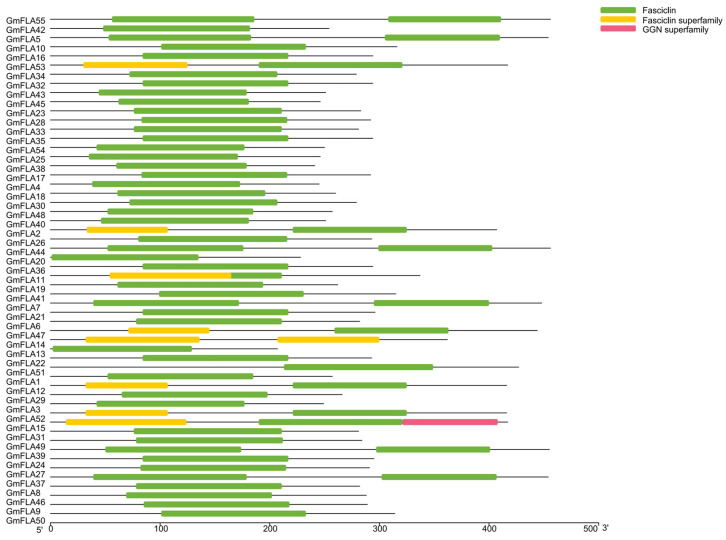
Conserved Domain of the Soybean *FLA* Gene Family.

**Figure 5:**
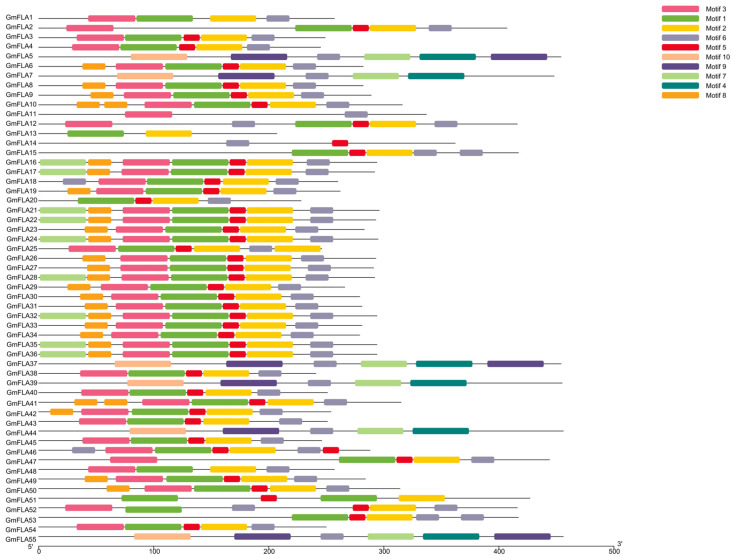
Conserved motif of the soybean *FLA* gene family.

**Figure 6:**
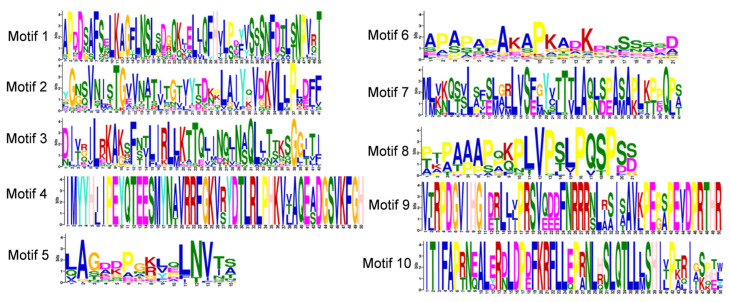
Amino acid sequences of 10 conserved motifs.

Analysis of conserved domains in soybean FLA proteins using the NCBI CD-Search tool revealed that all family members harbor a typical fasciclin domain (Figure 4), with highly conserved position and boundaries (average coverage > 95%). This domain persists stably across phylogenetic branches, confirming it as the core functional module of the soybean *FLA* family and suggesting strong functional constraints during evolution. Visual analysis of 10 conserved motifs (Motif 1–10) (Figure 5 and Figure 6) identified using MEME v5.5.2 revealed that Motif 1 is present in all soybean *FLA* members (100%). Its sequence localization overlaps with the core region of the Fasciclin domain, indicating this motif plays a crucial role in maintaining domain function. Secondary conserved motifs Motif 12 and Motif 3 exhibited frequencies of 92.1% and 85.3%, respectively, primarily distributed in the flanking regions of the Fasciclin domain; a repetition phenomenon occurred where Motif 6, Motif 8, and Motif 9 exhibit duplicate copies (each appearing twice) in 32.7% of members. These repeated units are predominantly located at the N/C-termini of proteins, suggesting potential involvement in regulating protein interactions or subcellular localization.

### 2.6. Analysis of Cis-Acting Elements in the Soybean FLA Gene Family

**Figure 7:**
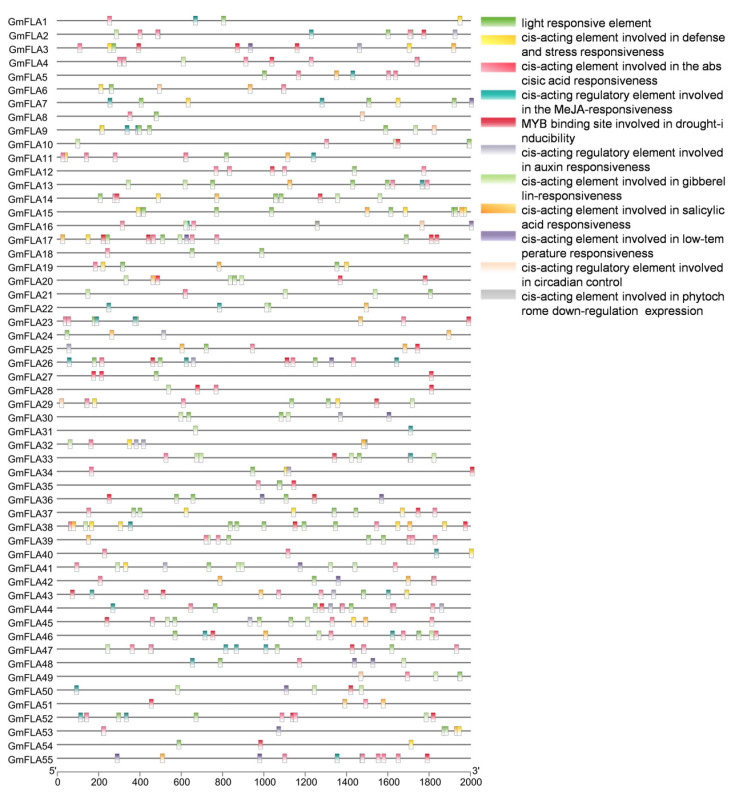
Soybean *FLA* Gene Family Cis-Acting Elements.

**Figure 8:**
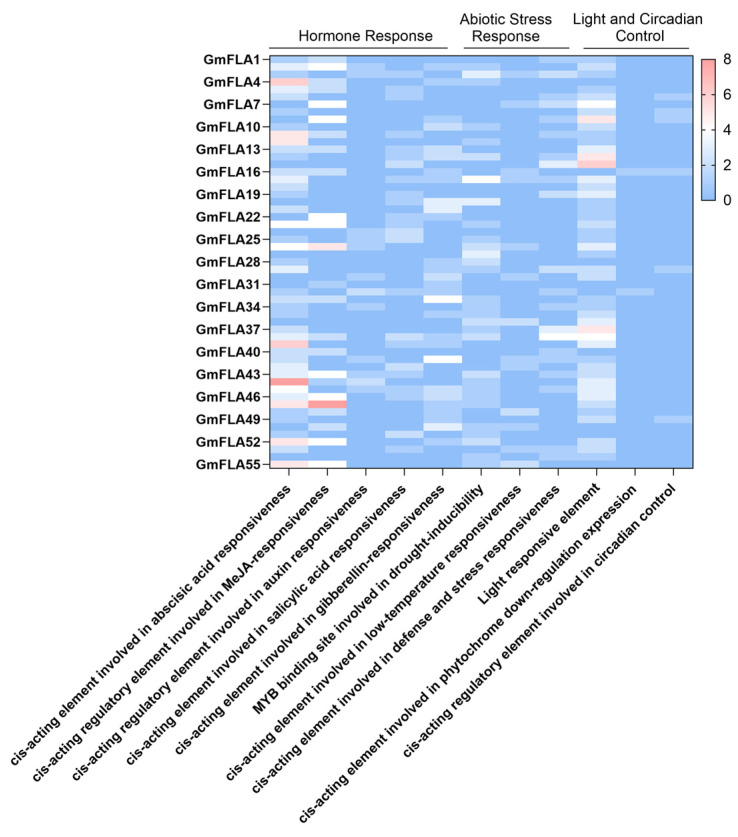
Cluster Analysis of Cis-Acting Elements in the Soybean *FLA* Gene Family.

Analysis of cis-acting elements in the promoter regions of the 55 GmFLA genes revealed that members of this family commonly harbor multiple elements associated with hormone response, abiotic stress, and growth and development (Figure 7 and Figure 8). Critically, we identified a high prevalence of key drought-responsive motifs, including the abscisic-acid-responsive element (ABRE, 88 total), dehydration-responsive elements (DRE, present in multiple members), and MYB binding sites (50 total), suggesting that GmFLA genes are likely involved in core drought and ABA signaling pathways in soybean.

Among other stress-related elements, defense and stress response elements totaled 37 (with GmFLA38 containing 4), and low-temperature response elements numbered 11 (GmFLA36 and GmFLA55 each contained 2). Hormone response elements were also widely distributed, with methyl jasmonate response elements (70), abscisic acid response elements (ABRE, 88), gibberellin response elements (45), and salicylic acid response elements (30) being the most abundant.

Furthermore, light-response elements (82 in total) and a small number of circadian clock regulatory elements were identified, indicating potential roles in light signal integration. GmFLA15, GmFLA44, and GmFLA47 were the primary carriers of light-response, ABRE, and MeJA-response elements, respectively. Overall, the diverse cis-element profile suggests that GmFLA family genes may participate in complex regulatory networks coordinating light signaling, hormone-mediated growth, and stress responses.

### 2.7. Preliminary Screening of the FLA Gene Family in Soybean for Drought Response

In the Gene Expression Omnibus (GEO) database, the “Search for Studies at GEO Datasets” tool was used to retrieve records using the keyword “drought stress”. The search was then filtered by species (soybean) and data expression type (“Expression profiling by array”), yielding 10 records. Five datasets (GSE102749, GSE65553, GSE50408, GSE429663, and GSE40604) were selected for subsequent analysis based on the following criteria: compatibility with the GEO2R analytical tool, inclusion of both biological replicates and appropriate control samples, and coverage of different stress durations and tissue types (roots and leaves). These datasets were analyzed using the online tool Analyze with GEO2R within the GEO database. Root and leaf expression data were processed separately to maintain tissue-specific resolution in the analysis of drought responses. Differentially expressed genes (DEGs) were screened based on *p* < 0.05 and |logFC| ≥ 1, ultimately identifying five genes (Table 2): *GmFLA*5, *GmFLA*15, *GmFLA*40, *GmFLA*47, and *GmFLA*54. The GenBank accession numbers and detailed information of these five genes are provided in Table S1 (see Supplementary Materials).

### 2.8. Expression Patterns of Candidate FLA Genes Under Different Stress Conditions

**Figure 9:**
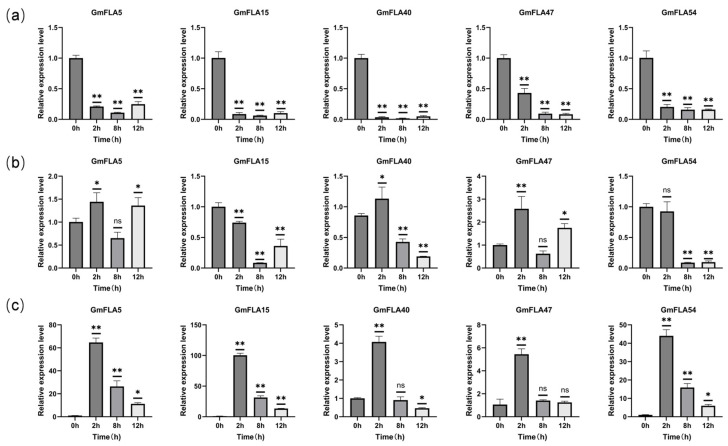
Tissue expression analysis of soybean candidate genes under drought stress: (**a**) root (**b**) stem (**c**) leaf * *p* < 0.05, ** *p* < 0.01, ns, Not significant.

Gene expression analysis was evaluated via quantitative real-time PCR (qRT-PCR). The expression of soybean *FLA* genes was analyzed in vegetative-stage seedlings following a 15% PEG6000-induced drought stress. Root, stem, and leaf tissues were sampled at 0, 2, 8, and 12 h post-treatment (*n* = 3 biological replicates). qRT-PCR revealed distinct temporal expression patterns among several FLA genes, demonstrating their activation during the early drought response (Figure 9).

The qRT-PCR results revealed that five candidate *GmFLA* genes (*GmFLA*5, *GmFLA*15, *GmFLA*40, *GmFLA*47, and *GmFLA*54) exhibited significant tissue-specific and time-dependent expression patterns under drought stress. In root tissues, the consistent downregulation of all five genes coincides with the typical drought-induced inhibition of root growth, potentially reflecting a strategic reallocation of resources from cell wall expansion to the maintenance of essential root functions involved in water uptake. Within stem tissues, the gene expression patterns diverged. The upregulation of *GmFLA*5, *GmFLA*40, and *GmFLA*47 suggests their potential involvement in stem mechanical reinforcement and vascular function maintenance under dehydration stress, while the sustained suppression of *GmFLA*54 may indicate its specific role is dispensable during stem stress adaptation. In leaves, all genes demonstrated the most rapid response, with strong induction observed at the 2 h stress time point. This rapid induction in leaves aligns with ABA-mediated drought responses, potentially contributing to cell wall modifications that facilitate stomatal regulation and water conservation under dehydration conditions. Although expression levels gradually declined with prolonged stress, they remained significantly elevated above baseline levels at 12 h. Notably, *GmFLA*54 exhibited a distinctive pattern, showing stable suppression in both roots and stems but strong induction in leaves, highlighting its unique organ-specific regulatory mechanism.

## 3. Discussion

This study successfully identified 55 *FLA* genes in the soybean genome, a number significantly higher than that in Arabidopsis (21) and rice (27). This finding aligns with two whole-genome duplication events in soybean history [15,16,17,34], suggesting lineage-specific expansion as the primary driver of *GmFLA* family evolution. Such expansion likely provided abundant genetic material for soybean adaptation to complex environments, such as drought stress and nodule development. The retention of a substantial number of *GmFLA* duplicates following polyploidization events may reflect selective pressures to maintain or refine specialized functions pertinent to soybean’s ecological adaptation and agronomic traits. Phylogenetic analysis further revealed that 82% of members clustered within a single major evolutionary branch, forming multiple soybean-specific lineages (Figure 1), strongly supporting the central role of gene duplication in family expansion. The emergence of these soybean-specific lineages suggests potential functional diversification or specialization within the *FLA* family, possibly contributing to unique aspects of soybean biology. Notably, this branch includes several *FLA* homologs known to be associated with stress responses, suggesting their functions could potentially be enriched toward environmental adaptation.

All *GmFLA*s contain a typical Fasciclin domain, with Motif 1 being highly conserved across all members and covering the core region of this domain. This feature aligns closely with the reported functions of FLAs in Arabidopsis and rice, which mediate protein–protein or protein–cell wall interactions [14,26], suggesting that *GmFLA*s may similarly participate in cell wall construction and regulation. Notably, the conserved fasciclin domain, often associated with cell adhesion, together with the characteristic AGP (arabinogalactan protein) motifs, could potentially enable these *GmFLA*s to potentially serve as molecular bridges linking the plasma membrane to the cell wall matrix, possibly participating in the perception and transmission of osmotic stress signals under drought conditions. Based on this structural feature, we hypothesize that specific *GmFLA*s might function as potential components in the cell wall–plasma membrane continuum, potentially modulating mechanical properties of cell walls and contributing to membrane integrity under dehydration stress. More specifically, they could potentially influence cell-wall stiffening by cross-linking wall components or through interactions with the cellulose–hemicellulose network, and might contribute to osmotic adjustment by affecting wall porosity and water-holding capacity.

Subcellular localization predictions suggest diverse localizations for *GmFLA*s, including chloroplasts (26 members), cell wall, vacuole, and plasma membrane. However, these computational predictions require cautious interpretation, as the actual localization of FLA proteins is known to be determined by complex post-translational mechanisms. Studies in Arabidopsis demonstrate that GPI anchoring and AG glycosylation collectively guide FLAs to the cell surface and subsequently to the cell wall [33]. This established mechanism aligns with our signal peptide analysis, which confirms that most *GmFLA*s contain signal peptides and are likely secreted to function primarily at the cell surface or within the cell wall matrix [8,9].

Promoter analysis revealed widespread cis-elements for light, ABA, and MeJA responses, with drought-induced MYB binding sites occurring most frequently. This provides a potential transcriptional regulatory basis for *GmFLA*s’ potential involvement in drought responses. The prevalence of ABA-responsive elements further implies that these *GmFLA*s might function within the abscisic acid signaling pathway, a central regulator of drought responses. Moreover, the frequent occurrence of MYB binding sites suggests a potential link to reactive oxygen species (ROS) signaling and cell wall integrity surveillance pathways, both of which are activated under drought stress and involve MYB transcription factors. This implies *GmFLA*s might function at the intersection of ABA signaling, ROS homeostasis, and cell wall dynamics under drought. These regulatory features support a functional hypothesis where drought-responsive *GmFLA*s may integrate ABA and ROS signaling pathways to coordinate cell wall remodeling with physiological responses to water deficit. Notably, members such as *GmFLA*38 and *GmFLA*44 contain multiple stress response elements, suggesting they might act as signal integration nodes. Their regulatory mechanisms could be further validated via yeast one-hybrid assays or ChIP-qPCR [22,28].

Through GEO data screening and qRT-PCR validation, this study identified five *GmFLA* genes (*GmFLA*5, *GmFLA*15, *GmFLA*40, *GmFLA*47, *GmFLA*54) exhibiting tissue-specific expression patterns under drought stress. Under drought stress, the *GmFLA* gene exhibits highly organ-specific expression, reflecting differentiated adaptive strategies adopted by different tissues in response to water deficit. The consistent downregulation of all genes in roots indicates an active metabolic conservation strategy, where resources are redirected toward critical survival mechanisms by suppressing gene expression associated with cell wall expansion. This root-specific downregulation, contrasting with upregulation in leaves, could reflect distinct tissue priorities: roots may reduce energy-intensive processes like cell wall remodeling to conserve resources, whereas leaves might prioritize cell wall reinforcement to limit water loss and maintain turgor. This coordinated downregulation in roots may represent a strategic reallocation of energy away from expansive growth and towards the reinforcement of existing cell walls, a process potentially mediated by specific FLAs, to enhance soil penetration and water foraging under dry conditions. We hypothesize that this transcriptional reprogramming might fine-tune root cell wall extensibility and mechanical strength, potentially influencing root system architecture and hydraulic conductance under water deficit. Differential expression patterns in stems reveal functional specificity: Upregulated genes likely contribute to maintaining vascular function and structural reinforcement. Conversely, the downregulation of *GmFLA*15 and *GmFLA*54 suggests their functions require suppression during stem stress adaptation. The rapid and robust induction of all genes in leaves strongly supports the critical role of FLA proteins in early leaf defense responses, such as stomatal closure and cell wall remodeling. This rapid induction in leaves is consistent with a putative role in cell wall remodeling to limit water loss and maintain tissue turgor, possibly through interactions with pectin and hemicellulose networks. Based on their domain characteristics and expression patterns, we propose that leaf-induced *GmFLA*s may modulate cell wall porosity and barrier properties, thereby regulating transpirational water loss while maintaining photosynthetic capacity under drought conditions. Particularly noteworthy are *GmFLA*15 and *GmFLA*54, which exhibit specific suppression patterns in roots and stems but are induced in leaves, making them promising candidates for studying organ-specific functional differentiation. Although this study, based on bioinformatics screening and partial gene validation, has not yet conducted systematic functional analysis of all 55 *GmFLA* members, its findings suggest the potential role of this family in drought response and provide candidate targets and hypotheses for subsequent functional studies using overexpression or silencing technologies.

Beyond the mechanistic insights, our findings possess broader biological and practical implications. The identified drought-responsive *GmFLA* genes, particularly those with tissue-specific induction patterns like *GmFLA*15 and *GmFLA*54 in leaves, represent promising candidates for molecular breeding. Their expression profiles could potentially serve as transcriptional markers for marker-assisted selection or biomarker development aimed at engineering drought-tolerant soybean varieties. While the functional conservation of these specific drought-responsive *GmFLA* genes across diverse soybean cultivars remains to be experimentally determined, our findings provide a theoretical foundation for improving drought tolerance in soybean. Future studies should investigate the variation and functional consistency of these candidate genes across different soybean germplasms. The potential applicability of these findings to other legume species would require identification and characterization of corresponding *FLA* homologs in those systems.

Future research should prioritize constructing overexpression, RNAi, or CRISPR-Cas9 mutant lines of key genes (e.g., GmFLA15, *GmFLA*54), combined with analyses of physiological indicators (water loss rate, photosynthetic efficiency) and cell wall components (pectin, hemicellulose), to systematically elucidate their specific mechanisms in drought resistance. Specifically, future work should test the proposed hypotheses by determining whether manipulating these candidate genes exerts effects on root architecture and leaf water retention precisely through modulating cell wall composition and mechanical properties. It is important to note that the PEG-simulated drought used in this study primarily induces osmotic stress and may not fully replicate field conditions; thus, field-based validation remains necessary. Furthermore, this study lacks protein-level validation. Future work should include Western blot analysis to confirm protein expression and glycosylation profiling to assess post-translational modifications, given their importance for AGP function. Glycoproteomics could be employed to investigate these regulatory mechanisms. Ultimately, field trials and cross-species comparisons will help comprehensively reveal the *FLA* family’s functional diversity in soybean environmental adaptation.

## 4. Materials and Methods

### 4.1. Identification of FLA Family Members in Soybean

The soybean genome data Glycine_max_v4.0 and its annotation files were downloaded from the NCBI Genome database. Subsequently, the following operations were performed sequentially using Tbtools (v2.0) software: (1) Extraction of soybean CDS sequences via the Gtf/Gff3 Sequences Extract function; (2) Simplification of sequence identifiers in FASTA files using the Fasta ID Simplify function; (3) Finally, the CDS sequences were translated into protein sequences using the Batch Translate CDS to Protein function.

To comprehensively identify *GmFLA* members, we employed a dual screening strategy: First, we performed BLASTP homology searches against the soybean proteome using all known Arabidopsis FLA protein sequences (downloaded from Arabidopsis databases) as query sequences, with an E-value threshold set to <1 × 10^−5^. Second, we performed searches using the Simple HMM Search function in TBtools (E-value < 1 × 10^−5^) based on the Hidden Markov Model (HMM, PF02469) for the Fasciclin conserved domain from the Pfam database. After merging and deduplicating candidate sequences from both methods, we rigorously validated each candidate online using the NCBI conserved domain database to eliminate false positives and ensure identification accuracy. Sequences that did not contain a complete, typical Fasciclin domain (as defined by the NCBI CDD with a domain E-value < 0.01 and coverage > 60%) were discarded. Only sequences confirmed to contain a complete, typical Fasciclin domain were ultimately designated as non-redundant members of the *FLA* gene family.

### 4.2. Chromosomal Localization and Physicochemical Properties Analysis of the Soybean FLA Gene Family

The TBtools Gene Location Visualize from Gtf/Gff function was used to visualize the chromosomal distribution of soybean *FLA* genes. The Protein Parameter Calc module was employed to calculate physicochemical parameters including molecular weight (Mw), isoelectric point (pI), instability coefficient, and hydrophilicity (GRAVY). Subcellular localization was predicted using WoLF PSORT (https://wolfpsort.hgc.jp/, accessed on 7 December 2024). Signal peptides were analyzed with SignalP 5.0 (https://services.healthtech.dtu.dk/service.php?SignalP-5.0, accessed on 7 December 2024) using a D-value threshold > 0.5. The integrated results were cross-validated to determine protein secretion mechanisms.

### 4.3. Analysis of Conserved Domains, Motifs, and Gene Structures in the Soybean FLA Gene Family

Soybean FLA protein sequences were submitted to the MEME online tool (https://meme-suite.org/meme/tools/meme, accessed on 9 December 2024) with 10 motifs and maximum length parameters. Motif Display in TBtools visualized the motifs, enabling comparison of motif distribution similarities among family members. Simultaneously, based on genomic GTF/GFF annotation files and CDS sequences, the intron–exon structure diagram was drawn using TBtools Gene Structure View (Advanced) function to systematically compare structural features among different genes.

### 4.4. Evolutionary Analysis and Cis-Acting Element Analysis of the Soybean FLA Gene Family

Arabidopsis *FLA protein* sequences were retrieved from the Arabidopsis database and merged with soybean *FLA* sequences into a single file. A phylogenetic tree was constructed using the Neighbor-Joining method in MEGA 11 software. Key parameters included 1000 bootstrap replicates to assess branch reliability, with other parameters set to default values. The final tree underwent topology optimization, annotation refinement, and publication-grade visualization adjustments via the iTOL online platform (https://itol.embl.de/, accessed on 9 December 2024).

Using the “Gtf/Gff3 Sequences Extract” and “Fasta Extract” functions in Tbtools (v2.0) software, extract promoter sequences spanning 2000 bp upstream of the transcription start site (TSS) for all *FLA* genes from the soybean reference genome; Submitted the obtained sequences to the PlantCARE database (https://bioinformatics.psb.ugent.be/webtools/plantcare/html/, accessed on 26 July 2025) for cis-acting element scanning and functional annotation; Based on biological function, key regulatory elements for abiotic stress responses (e.g., drought, cold, heat stress) and hormone responses (e.g., abscisic acid, methyl jasmonate) were retained. Visual analysis of the selected element types and their distribution patterns was performed using TBtools.

### 4.5. Screening of Drought Response Candidate Genes

We retrieved drought-related transcriptomic datasets from the NCBI GEO database by searching with the keyword “drought stress” and applying filters for the species (*G. max*) and data type (expression profiling by array). The dataset selection criteria required that studies: (1) include both biological replicates (at least two per condition) and suitable control samples (well-watered conditions); (2) provide coverage of key tissues involved in drought response, specifically roots and leaves. All differential expression analyses were performed using the GEO2R platform, which automatically executes background correction and normalization specific to each platform (as described in the GEO2R documentation). Differential expression analysis between drought-treated and control groups was conducted using GEO2R’s default parameters on the normalized expression values, with thresholds set at |logFC| ≥ 1 and an adjusted *p*-value (adj.*p*.Val) < 0.05. For probe annotation, we used the latest platform annotation files (GPL) provided by NCBI GEO without further updates. Finally, the resulting list of differentially expressed genes (DEGs) was cross-referenced with our pre-identified soybean FLA gene set to screen for drought-responsive candidates.

### 4.6. Plant Materials and Treatment Methods

Soybeans used in this study were provided by our laboratory. Soybean seeds were sown in soil and cultivated under controlled conditions (25 °C, 16 h light/8 h dark) until the vegetative growth stage, specifically the growth phase between the first and second trifoliate leaves. Irrigation with a 15% PEG6000 solution was employed to simulate drought stress. This treatment leveraged PEG6000’s high molecular weight to prevent penetration through cell walls, thereby inducing a water deficit state within plants that closely mimics natural drought conditions [35]. This approach facilitated the maintenance of stable and controllable stress conditions.

To dynamically monitor the early transcriptional response to drought stress, root, stem, and leaf samples were randomly collected at 0, 2, 8, and 12 h post-stress application. Each time point comprised three biological replicates, with each replicate derived from pooled tissues of different plants. Samples were immediately flash-frozen in liquid nitrogen and stored at –80 °C for subsequent gene expression analysis.

### 4.7. RNA Extraction and Quantitative Reverse Transcription Polymerase Chain Reaction (qRT-PCR)

RNA was extracted using the Plant Total RNA Extraction Kit from Fuji Bio Technology Co., Ltd. (Chengdu, China). cDNA synthesis was performed using the All-In-One 5X RT MasterMix with gDNA Removal Kit (Applied Biological Materials Inc., Richmond, BC, Canada) under the following conditions: 37 °C for 15 min, 60 °C for 10 min, and 95 °C for 3 min. Real-time quantitative PCR was performed on the LightCycler 96 system (Roche, Mannheim, Germany). The reaction mixture contained 10 μL TB Green^®^ Premix Ex Taq™ II, 0.8 μL each of forward and reverse primers, 2 μL cDNA template, and 6.4 μL ddH_2_O. Gene-specific primers were designed using the NCBI Primer-BLAST tool (https://www.ncbi.nlm.nih.gov/tools/primer-blast/, accessed on 20 December 2024) to ensure specificity, and the primer sequences used in this study are listed in Table S2 (see Supplementary Materials). The amplification specificity of each primer pair was validated by the appearance of a single peak in the melting curve analysis. The relative expression levels of target genes were calculated using the 2^−ΔΔCt^ method and normalized against the stable reference gene *CYP*2 (NC_038248).

## Figures and Tables

**Table 1:** Physicochemical Characterization of the *FLA* Gene Family in Soybean.

Gene Name	Gene ID	AA	MW (kDa)	pI	II	AI	GRAVY	Location	SP
*GmFLA*1	Glyma.02G307700	256	27,139.59	5.03	51.84	86.17	0.134	vacu	Yes
*GmFLA*2	Glyma.02G308700	406	43,557.75	6.37	36.8	91.16	−0.002	vacu	Yes
*GmFLA*3	Glyma.03G179900	248	26,301.83	5.51	26.81	88.91	0.083	chlo	Yes
*GmFLA*4	Glyma.03G180000	244	25,780.24	6.82	21.61	90.82	0.052	chlo	Yes
*GmFLA*5	Glyma.03G204300	453	50,149.18	5.98	58.21	94.97	−0.235	cyto	Yes
*GmFLA*6	Glyma.05G161900	281	29,174.37	9.12	41.47	95.59	0.093	chlo	Yes
*GmFLA*7	Glyma.06G308600	447	49,665.66	6.36	54.31	89.69	−0.287	extr	Yes
*GmFLA*8	Glyma.08G119400	281	29,247.45	9.14	44.82	97.3	0.095	extr	Yes
*GmFLA*9	Glyma.08G119500	288	29,955.41	9.34	44.01	97.67	0.111	plas	Yes
*GmFLA*10	Glyma.08G119600	315	32,662.67	8.47	45.63	98.76	0.124	chlo	Yes
*GmFLA*11	Glyma.08G183700	336	36,952.17	5.92	72.04	89.17	−0.035	chlo	No
*GmFLA*12	Glyma.08G329900	415	43,692.78	5.81	37.53	93.01	−0.054	plas	Yes
*GmFLA*13	Glyma.09G048100	206	21,492.33	4.53	42.49	93.2	0.178	golg	No
*GmFLA*14	Glyma.09G119800	361	39,979.15	6.53	40.55	87.59	−0.168	chlo	Yes
*GmFLA*15	Glyma.09G267700	416	42,505.2	5.59	48.51	97.45	0.189	plas	Yes
*GmFLA*16	Glyma.10G245800	293	31,032.8	8.82	43.86	99.52	0.063	vacu	Yes
*GmFLA*17	Glyma.11G147800	291	30,785.5	9.03	36.87	95.88	0.066	chlo	Yes
*GmFLA*18	Glyma.11G148100	259	27,402.34	7.96	33.06	92.32	0.036	chlo	Yes
*GmFLA*19	Glyma.11G148200	261	27,944.04	8.62	40.07	100.15	0.136	extr	Yes
*GmFLA*20	Glyma.11G148300	227	24,078.73	6.5	33.24	109.12	0.263	golg	No
*GmFLA*21	Glyma.11G148400	295	30,969.58	8.51	44.79	96.58	0.047	chlo	Yes
*GmFLA*22	Glyma.11G148500	292	30,793.46	9.2	45.12	94.9	0.02	chlo	Yes
*GmFLA*23	Glyma.11G148501	282	29,640.22	8.67	50.13	104.15	0.083	chlo	Yes
*GmFLA*24	Glyma.11G148700	294	30,890.6	8.53	45.22	97.18	0.097	vacu	Yes
*GmFLA*25	Glyma.11G155886	245	25,833.58	7.85	30.4	98.65	0.027	chlo	No
*GmFLA*26	Glyma.11G156304	292	30,731.29	8.93	39.59	90.79	0.041	chlo	Yes
*GmFLA*27	Glyma.11G156513	290	30,551.27	6.74	38.19	103.97	0.163	chlo	Yes
*GmFLA*28	Glyma.12G068900	291	30,717.5	9.24	39.58	99.9	0.089	chlo	Yes
*GmFLA*29	Glyma.12G069400	265	28,023.12	7.73	37.85	105.25	0.182	vacu	Yes
*GmFLA*30	Glyma.12G069500	278	29,334.82	6.84	52.07	100.04	0.094	extr	Yes
*GmFLA*31	Glyma.12G069601	280	29,451.91	8.69	50.82	98.96	0.093	extr	Yes
*GmFLA*32	Glyma.12G069700	293	30,964.66	7.81	48.53	96.55	0.027	chlo, vacu	Yes
*GmFLA*33	Glyma.12G069800	280	29,437.89	8.69	52.19	98.29	0.085	extr	Yes
*GmFLA*34	Glyma.12G069900	278	29,347.87	7.88	54.31	100.04	0.094	chlo, extr	Yes
*GmFLA*35	Glyma.12G070000	293	30,998.68	7.81	48.53	95.22	0.024	chlo, vacu	Yes
*GmFLA*36	Glyma.12G070200	293	30,936.6	7.81	48.24	95.9	0.019	chlo	Yes
*GmFLA*37	Glyma.12G096300	453	50,393.5	6.58	56.69	88.9	−0.331	vacu	Yes
*GmFLA*38	Glyma.12G173800	240	25,483.09	9.22	28.99	97.54	0.051	chlo	Yes
*GmFLA*39	Glyma.12G190700	454	50,214.24	6.13	52.12	92.11	−0.261	vacu	Yes
*GmFLA*40	Glyma.12G207600	250	26,345.18	9.19	31.43	88.6	−0.078	extr	Yes
*GmFLA*41	Glyma.13G226000	314	32,532.23	8.73	44.52	91.05	0.072	chlo	Yes
*GmFLA*42	Glyma.13G226100	253	26,568.34	9.48	37.27	91.82	−0.022	chlo	No
*GmFLA*43	Glyma.13G293500	250	26,589.38	8.56	31.46	93.28	−0.109	extr	Yes
*GmFLA*44	Glyma.13G311000	455	50,328.36	6.2	53.1	91.93	−0.264	chlo	Yes
*GmFLA*45	Glyma.13G327100	245	26,280.28	9.56	27.45	94.37	0.052	chlo	Yes
*GmFLA*46	Glyma.13G327300	287	29,843.57	9.25	33.23	102.93	0.208	plas	Yes
*GmFLA*47	Glyma.14G004200	443	47,882.64	7.29	41.38	89.73	−0.077	plas	Yes
*GmFLA*48	Glyma.14G005300	256	27,224.66	5.05	47.17	85.04	0.102	plas	Yes
*GmFLA*49	Glyma.15G086000	283	29,817.32	9.44	37.12	101.06	0.113	chlo	Yes
*GmFLA*50	Glyma.15G086100	313	32,586.38	9.22	46.8	91.02	0.036	chlo	Yes
*GmFLA*51	Glyma.15G155400	426	45,166.65	5.68	45.51	105.77	0.286	plas	Yes
*GmFLA*52	Glyma.18G076600	415	43,368.38	5.8	34.03	93.71	−0.021	plas	Yes
*GmFLA*53	Glyma.18G222200	416	42,488.34	5.89	47.02	98.37	0.18	plas	Yes
*GmFLA*54	Glyma.19G180700	249	26,278.71	5.51	26.22	90.92	0.074	plas	Yes
*GmFLA*55	Glyma.19G201600	455	50,578.56	6.21	61.05	90.68	−0.308	cyto	Yes

**Table 2:** Differentially Expressed Gene Screening Results.

Dataset	Group	Fold Change				
		*GmFLA*5	*GmFLA*15	*GmFLA*40	*GmFLA*47	*GmFLA*54
GSE102749	water control—5 h water stress (root)	0.2393	0.5564	0.9766	0.1159	0.0855
	water control—48 h water stress (root)	−0.18	−0.0293	−0.287	0.162	−0.927
GSE6553	water control—2 h water stress (root)	0.571	0.5026	0.7178	−0.0241	0.7653
	water control—10 h water stress (root)	0.855	0.848	0.835	0.0888	0.799
GSE49537	water control—30 min water stress (root)	0.1684	0.5878	0.4536	0.4040	0.3544
	water control—2 h water stress (root)	0.0947	0.4811	2.1924	0.5313	0.2129
	water control—5 h water stress (root)	0.6182	1.6081	1.7033	1.0337	1.6471
	water control—30 min water stress (leaf)	0.3239	1.3927	−0.2175	0.2456	−0.1039
	water control—2 h water stress (leaf)	1.5716	2.5599	2.0871	1.2721	0.6380
	water control—5 h water stress (leaf)	2.7291	5.0547	5.4540	2.4745	2.5847
GSE29663	water control—6 d water stress (leaf)	0.2191	0.0645	2.4010	1.2771	1.0363
GSE40604	water control—5 d water stress (leaf)	1.5291	−0.0531	0.9784	1.2180	0.5417

## Data Availability

All data are displayed in the manuscript and Supplementary Materials.

## Supplementary Materials

The following supporting information can be downloaded at: https://www.mdpi.com/article/10.3390/genes16121425/s1, Table S1: Information on the five FLA family genes identified from GEO datasets under drought stress in soybean. Table S2: Primer sequences for qRT-PCR analysis of the five FLA genes.
