# Supplementary material for: Identification of the FLA Gene Family in Soybean and Preliminary Functional Analysis of Its Drought-Responsive Candidate Genes
Source: Genes (Basel). 2025 Nov 29;16(12):1425. doi: 10.3390/genes16121425 (PMC12732659; doi:10.3390/genes16121425)
Supplement: Supplementary file 1 [file genes-16-01425-s001.zip › Table S1—— Information on the five FLA family genes identified from GEO datasets under drought stress in soybean..pdf]

Gene name	Gene symbol	Gene ID	Gene Locus ID
GmFLA5	LOC100800956	100800956	Glyma.03G204300
GmFLA15	LOC100787866	100787866	Glyma.09G267700
GmFLA40	LOC100500293	100500293	Glyma.12G207600
GmFLA47	LOC100799605	100799605	Glyma.14G004200
GmFLA54	LOC100805219	100805219	Glyma.19G180700
