# Supplementary material for: Identification of the FLA Gene Family in Soybean and Preliminary Functional Analysis of Its Drought-Responsive Candidate Genes
Source: Genes (Basel). 2025 Nov 29;16(12):1425. doi: 10.3390/genes16121425 (PMC12732659; doi:10.3390/genes16121425)
Supplement: Supplementary file 1 [file genes-16-01425-s001.zip › Table S2——Primer sequences for qRT-PCR analysis of the five FLA genes..pdf]

Supplementary Documents

Primer Name	Sequence（5’→3’）	Purpose
qGmFLA15-F	TTCTCCTACTCCTGCACCGT	Fluorescent Quantitative Primers
qGmFLA15-R	CTTACCGGCGGAACTACTCC	
qGmFLA40-F	GGTGAACATCTCCACAGGGG	
qGmFLA40-R	GTTCCAACGGCAACAGAACC	
qGmFLA47-F	ACAAGAACAACAGCCATGCG	
qGmFLA47-R	GAATCTCACGTGCAATCCGC	
qGmFLA54-F	ATGAAGCTTTTCGGCGCAAC	
qGmFLA54-R	GTGTCCGACGACCCTGAAG	
qGmFLA5-F	GACCGTCCATCGCTCCTG	
qGmFLA5-R	TGAAGTCCTTCACCTGACGC	
CYP2-F	CGGGACCAGTGTGCTTCTTCA	Soybean Internal Reference Gene Primers
CYP2-R	CCCCTCCACTACAAAGGCTCG	
